# A Review on Drug Delivery System for Tumor Therapy

**DOI:** 10.3389/fphar.2021.735446

**Published:** 2021-10-04

**Authors:** Guoxiang Liu, Lina Yang, Guang Chen, Fenghua Xu, Fanghao Yang, Huaxin Yu, Lingne Li, Xiaolei Dong, Jingjing Han, Can Cao, Jingyu Qi, Junzhe Su, Xiaohui Xu, Xiaoxia Li, Bing Li

**Affiliations:** ^1^ Department of Genetics and Cell Biology, Basic Medical College, Qingdao University, Qingdao, China; ^2^ Department of Hematology, The Affiliated Hospital of Qingdao University, Qingdao, China

**Keywords:** drug delivery system, delivery carriers, antitumor drug, targeting, tumor therapy

## Abstract

In recent years, with the development of nanomaterials, the research of drug delivery systems has become a new field of cancer therapy. Compared with conventional antitumor drugs, drug delivery systems such as drug nanoparticles (NPs) are expected to have more advantages in antineoplastic effects, including easy preparation, high efficiency, low toxicity, especially active tumor-targeting ability. Drug delivery systems are usually composed of delivery carriers, antitumor drugs, and even target molecules. At present, there are few comprehensive reports on a summary of drug delivery systems applied for tumor therapy. This review introduces the preparation, characteristics, and applications of several common delivery carriers and expounds the antitumor mechanism of different antitumor drugs in delivery carriers in detail which provides a more theoretical basis for clinical application of personalized cancer nanomedicine in the future.

## Introduction

According to global cancer statistics, there were 19.3 million new cancer cases and 10 million deaths in 2020 (Sung et al., 2021). The research of cancer treatment is imperative. The most common methods of cancer treatment include surgery, radiotherapy, chemotherapy, and targeting therapy (Wei et al., 2017; Mun et al., 2018). First of all, the removal of tumor mass by surgery is the basic treatment for most cancer patients; Secondly, radiotherapy and chemotherapy, as traditional cancer treatment tools, have outstanding effects in inhibiting the rapid growth of tumors (Mitra et al., 2015). Clinical data have shown that surgical resection in combination with radiation therapy have been a standard strategy for primary tumors without obvious metastasis. However, in view of many strongly aggressive tumors such as malignant peripheral nerve sheath tumors and metastatic melanoma with a high fatality rate, the limitations of these methods are obvious in patients and could result in poor outcomes and high relapse rates (Bishop et al., 2018; Tian et al., 2021). In order to improve the cure and survival rate of cancer patients, the development and application of multiple drug delivery systems as novel and more promising methods are widely used in cancer treatment. For example, pegylated liposomal doxorubicin (DOX; Doxil^®^/Caelyx^®^) and nab–paclitaxel (PTX) (Abraxane^®^) as first-generation nanomedicine drugs based on passive targeting by modulating their physicochemical properties had been used in clinical practice. Interestingly, the second generation of nanomedicine drugs based on active targeting has become a research hotspot of drug delivery systems, which further opens up a new way for the clinical treatment of cancer (Wicki et al., 2015). Targeting ligands such as peptides (Liu et al., 2014), small organic molecules (Sahu et al., 2011; Narmani et al., 2019; Mansoori et al., 2020), and antibodies (Julien et al., 2011) have been added to the surface of NPs selectively binding to overexpressed targeting receptors of certain tumor cells. Then, antitumor drugs can kill tumor cells through delivery carriers (Sun et al., 2014; Wicki et al., 2015). In several preclinical experiments, second generation of nanomedicine drugs such as immunoliposomes (ILs), including anti-HER2-ILs, anti–epidermal growth factor receptor–ILs (anti-EGFR-ILs), anti-VEGFR2-ILs, and other antibodies-ILs have been developed. The anti-EGFR antibody (cetuximab) was conjugated to DOX-loaded pegylated liposomes to evaluate the curative effect on patients with advanced solid tumors (Wicki et al., 2015). However, lots of developed drug delivery systems, especially the second generation of nanomedicine drugs for active targeted therapy, have been used in early clinical trials, and only a few of them have been approved for commercial use. In this review, we summarize the development and application of drug delivery systems, aiming to achieve effective delivery of antitumor drugs and their clinical application in the future.

## Preparation, Characteristics, and Application of Several Common Drug Delivery Carriers

In recent years, many inorganic materials (nonmetallic and metal) and organic materials (natural polymers, liposomes, exosomes, and dendrimer) have been studied as delivery carriers and designed into multifunctional drug delivery systems with the best size, shape, and surface properties to optimize the antitumor effect (Chen et al., 2013; Sun et al., 2014; Baetke et al., 2015; Wicki et al., 2015; Thomas et al., 2019).

### Nonmetallic NPs

Silicon and carbon are the most significant nonmetallic substances in close acquaintance with human life. Due to their intrinsic physical/chemical properties, advantages of low cost, and high biocompatibility, they can be used to make nanocarriers for cancer diagnosis and treatment (Chen et al., 2013). In recent years, nonmetallic NPs such as silicon NPs (SiNPs), porous SiNPs (PSiNPs), graphene, and graphene oxide (GO) have emerged as a new area especially for the development of drug delivery systems for cancer treatment.

**FIGURE 1:**
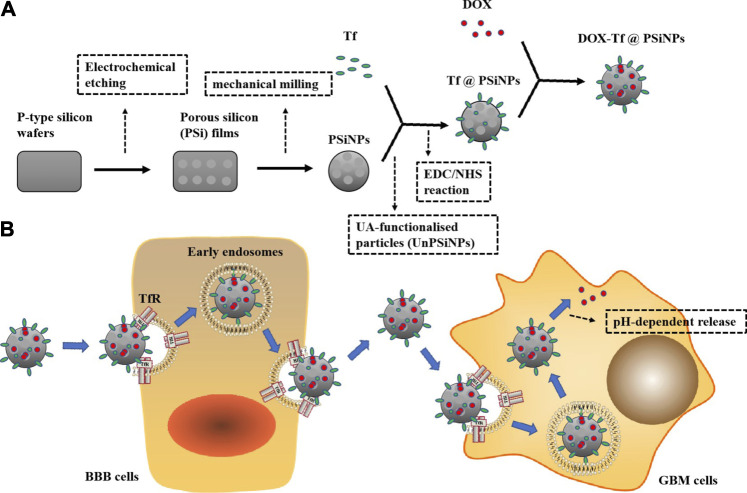
Construction, uptake, release, and efficacy of DOX-Tf@PSiNPs. **(A)** P-type silicon wafers are used to prepare porous silicon (PSi) films by electrochemical etching, and PSiNPs are formed by mechanical milling. UA-functionalized PSiNPs (UnPSiNPs) conjugate Tf through a two-step EDC/NHS reaction, and further loading DOX to form DOX-Tf@PSiNPs. **(B)** Nanoparticles can be combined with TfR through Tf to achieve targeting ability across the BBB into GBM cells, and release DOX in a pH-dependent way.

SiNPs are prepared by femtosecond laser ablation in deionized water. SiNPs have lots of advantages in cancer treatment, including biocompatibility, biodegradability, low cytotoxicity, and genotoxicity, and can be completely degraded by cells and tissues. In addition, SiNPs can also provide photodynamic therapy and radiofrequency hyperthermia for cancer therapy, due to the characteristics of room temperature photoluminescence, singlet oxygen generation under photoexcitation, and infrared radiation-induced and ultrasound-induced hyperthermia. PSiNPs produced by mechanical milling of electrochemically prepared porous silicon have biocompatibility, biodegradability, high drug loading capacity, and versatile surface modifications. They can be used as dissolved nano-containers which offer a platform for the vectorization of hydrophobic drugs with high quantities into the pores while allowing the immobilization of the targeting molecule on the surface of the nanoparticles (NPs) (Tamarov et al., 2014; Tolstik et al., 2016). PSiNPs have exhibited potential applications for cancer theranostics, such as tumor imaging, chemotherapy, photodynamic therapy, gene therapy, immunotherapy, and targeted therapy (Landgraf et al., 2020; Xia et al., 2018). As shown in Figure 1, transferrin (Tf)-conjugated PSiNPs are loaded with DOX as a new type of glioblastoma multiforme (GBM)–targeting NPs (DOX-Tf@PSiNPs), showing high drug loading ability. The NPs can be combined with overexpressed Tf receptors (TfRs) on the surfaces of the blood–brain barrier (BBB) and GBM cells, enhancing the internalization efficiency under clathrin-mediated endocytosis, and controlling the intracellular release of DOX. In the *in vitro* BBB model, compared to free DOX, DOX-Tf@PSiNPs displays higher cytotoxicity to GBM cells across the BBB (Luo et al., 2019).

**FIGURE 2:**
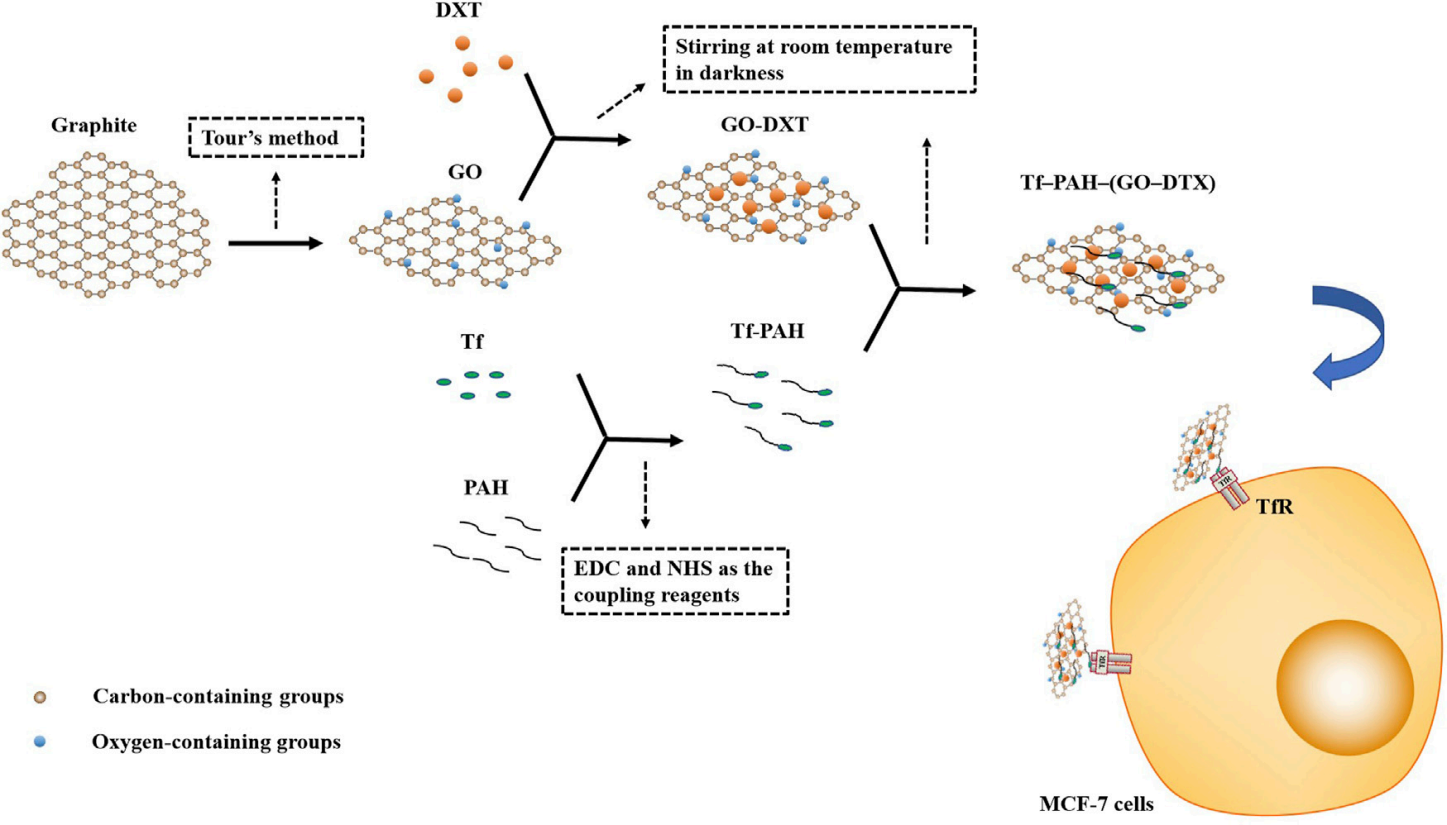
Preparation of targeted GO. GO is prepared from graphite through the tour method. The mixture of GO and DXT was stirred for 24 h at room temperature in darkness to prepare GO-DXT. Tf-PAH is synthesized through the formation of amide linkages between COOH groups of Tf and NH2 groups of PAH through EDC and NHS as the coupling reagents. Tf-PAH-(GO-DTX) is prepared by stirring the mixture of GO-DXT and Tf-PAH for 10 h at room temperature in darkness. Tf-PAH-(GO-DTX) could be taken up into MCF-7 cells by targeting TfR which would exert toxic effects.

Graphene and GO are synthesized via the Hummers' method (Wu et al., 2015). Graphene, a single layer of sp2-hybridized carbon atoms arranged in a honeycomb two-dimensional (2-D) crystal lattice, consists of a layer with a p-conjugated structure of six atom rings, which can be conceptually viewed as a planar aromatic macromolecule (Feng et al., 2013; Liu et al., 2013; Mousavi et al., 2019). Graphene, its oxidized form named as GO, has a mixed structure equipped with various oxygen-containing functional groups (epoxy, hydroxyl, carboxylic, carbonyl, etc.) and provides attachment sites to various biological molecules including protein, DNA/RNA, etc. (Singh DP. et al., 2018). Due to their unique properties, e.g., 2-D planar structure, large surface area, chemical and mechanical stability, superb conductivity, and good biocompatibility, graphene and GO have been extensively highlighted as promising biomaterials for biomedical applications, including biosensing, bioimaging, drug and gene delivery, and photothermal therapy (PTT) in cancer treatment. In particular, they have been extensively studied as drug delivery systems for loading anticancer drugs (Mousavi et al., 2019; Zhou et al., 2018). In Figure 2, GO-based nanocarriers could deliver potent hydrophobic docetaxel (DTX) by functionalizing with Tf-poly (allylamine hydrochloride) (PAH), which provide targeted and specific accumulation to TfR. Compared to nontargeted carrier and free DTX, Tf-PAH-(GO-DTX) as a promising nanocarrier could be taken up into MCF-7 cells in higher amounts, which enhanced significantly higher cytotoxicity of DTX (Nasrollahi et al., 2016).

### Metal NPs

**FIGURE 3:**
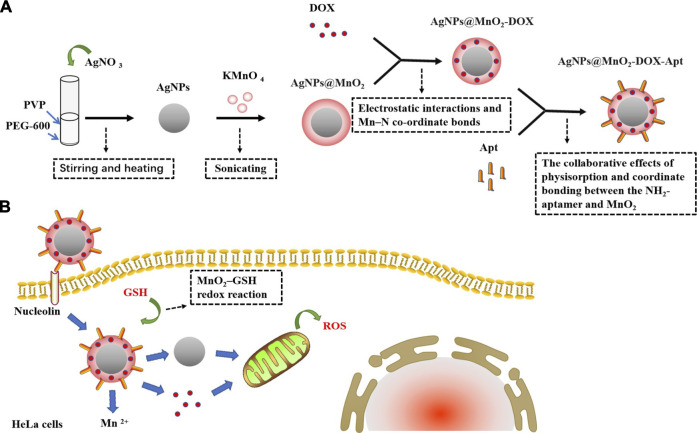
The synthesis of AgNPs@MnO_2_-DOX-Apt and pro-apoptotic mechanism. **(A)** AgNO_3_ aqueous solution is added into the mixed liquor of PEG-600 and PVP, and then AgNPs are synthesized under the conditions of stirring and heating. The AgNPs mixture added with KMnO_4_ is sonicated to synthesize AgNPs@MnO_2_ core–shell nanostructure for MnO_2_-modified AgNPs, and the MnO_2_ nanosheets can load DOX via electrostatic interactions and Mn–N co-ordinate bonds. Finally, AgNPs@MnO_2_-DOX-Apt is synthesized by functionalizing the Apt onto the MnO_2_ nanosheets via the collaborative effects of physisorption and coordinates bonding between the NH2-Apt and MnO_2_. **(B)** AgNPs@MnO_2_-DOX-Apt can enter HeLa cells through nucleolin receptor-mediated endocytosis, and release AgNPs and DOX under the response of MnO_2_–GSH redox reaction which could induce ROS-mediated apoptosis.

As we all know, metals are natural elements on the Earth, and their applications exist in various fields, such as industry, agriculture, medicine, and our daily life. In terms of nanomaterials, metals have also been used to synthesize metal NPs (MtNPs) (Durán et al., 2016; Hurtado-Gallego et al., 2019). AgNPs, AuNPs, ZnO NPs, Fe_2_O_3_ NPs, CuO NPs, and Al_2_O_3_ NPs are the most common materials used to synthesize MtNPs by mechanical attrition, laser ablation, photo reduction, chemical electrolysis, and synthesis by organisms such as bacteria and plants (Ahmad et al., 2010; Hanan et al., 2018; Ovais et al., 2018a; Rao et al., 2016; Thakkar et al., 2010). MtNPs, not only have their own characteristic physicochemical properties but also contain antimicrobial, anticancer, catalyzing, optical, electronic, and magnetic properties; have been widely applied in different areas, such as biology, food, agriculture, engineering, electronics, cosmetics, and medicine; and have also been used in food and biomedical devices (Ovais et al., 2018a). Studies have shown that AgNPs and AuNPs are widely used in cancer research because of their cytotoxicity (Pan et al., 2009; Rao et al., 2016). In addition to being used alone, these NPs can also combine with biomolecular substances such as peptides, antibodies, and DNA/RNA or oligonucleotides, etc. (Kumar et al., 2015; Sharma et al., 2018; Zhou et al., 2016). In Figure 3, Zhou et al. (2016) studied AgNPs@MnO_2_-DOX-Apt nanoprobes based on DOX-loaded AgNPs@MnO_2_ modified with the AS1411 (an antiproliferative G-rich phosphodiester oligonucleotide) aptamer (Apt) which could target nucleolin on the surface of HeLa cells. After being taken up into the cells, AgNPs@MnO_2_-DOX-Apt could release AgNPs and DOX through glutathione (GSH) reducing MnO_2_ into Mn^+2^. The study showed that both AgNP and DOX could induce cytotoxicity and verified that the toxicity was mainly caused by reactive oxygen species (ROS)–mediated apoptosis from mitochondria. Compared with AgNPs@MnO_2_-Apt and MnO_2_-DOX-Apt, AgNPs@MnO_2_-DOX-Apt had a stronger therapeutic effect due to the synergism of AgNPs and DOX (Zhou et al., 2016).

**FIGURE 4:**
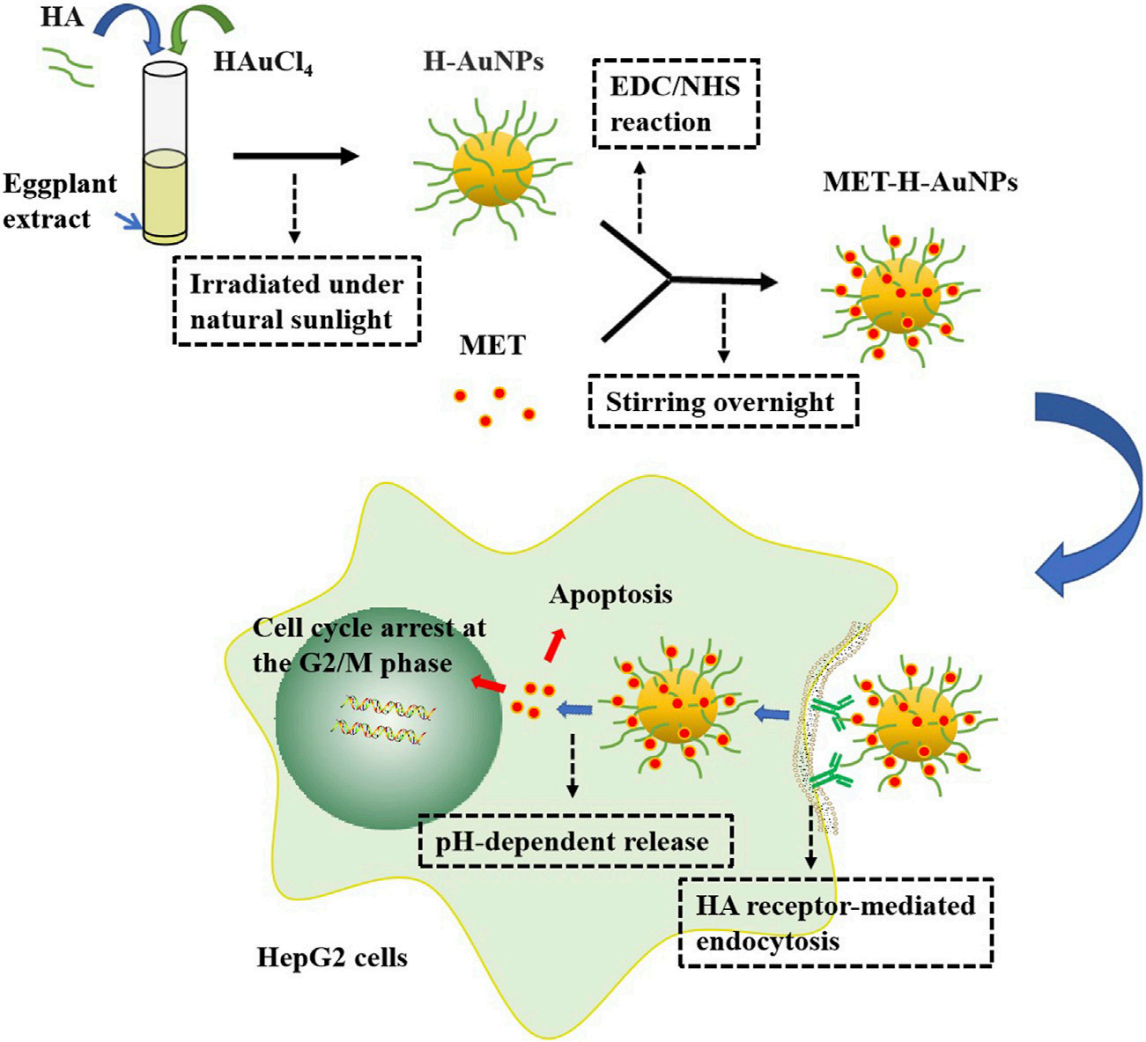
Synthesis of MET-H-AuNPs and targeted antitumor pathway. H-AuNPs are synthesized with the mixture of HAuCl4, HA, and eggplant extract irradiated under natural sunlight. After being modified by EDC/NHS reaction, H-AuNPs connect MET with the amide bond formed by stirring overnight between carboxyl groups of HA and amine group of MET to generate MET-H-AuNPs. MET-H-AuNPs are taken up into HepG2 cells through HA receptor–mediated endocytosis and release MET in a pH-dependent way which could induce cell cycle arrest significantly at the G2/M phase and apoptosis.

In biomedical research, due to high energy consumption and low yield of physical synthesis and the participation of toxic solvents and formation of harmful by-products in chemical synthesis, biologically synthesized (green) MtNPs showed advantages being economic, biocompatible, nontoxic, and environmentally friendly for bioactive plant compounds to improve these deficiencies (Hanan et al., 2018; Mukherjee et al., 2013; Mukherjee et al., 2015; Ovais et al., 2018a; Ovais et al., 2018b; Patra et al., 2015; Thakkar et al., 2010). Kumar et al. (2015) achieved green synthesis of AuNPs using the extract of the eggplant as a reducing agent. MET-H-AuNPs were synthesized with MET-loaded on HA-capped AuNPs (H-AuNPs) which could target HA receptors such as the hyaluronan receptor for endocytosis and the cluster determinant CD44 of HepG2 cells by HA and exhibited better cytotoxic activity than free MET. MET-H-AuNPs regulated the cell cycle by inhibiting the expression of Cyclin D1 and Cyclin E and induced apoptosis by upregulating pro-apoptotic proteins Bax, Caspase 9, Caspase 3, and PARP proteins and downregulating anti-apoptotic proteins Bcl-2, CDK-4, CDK-6, and CDK-2. The NPs could also increase the expression of p53 and induce cell apoptosis. This mechanism of MET-H-AuNPs provides a theoretical basis for clinical application in the future (Figure 4).

### Natural Polymer NPs

Glycan (chitosan) or protein (albumin and ferritin) as natural molecular materials have been often used to construct delivery carriers (Wicki et al., 2015).

**FIGURE 5:**
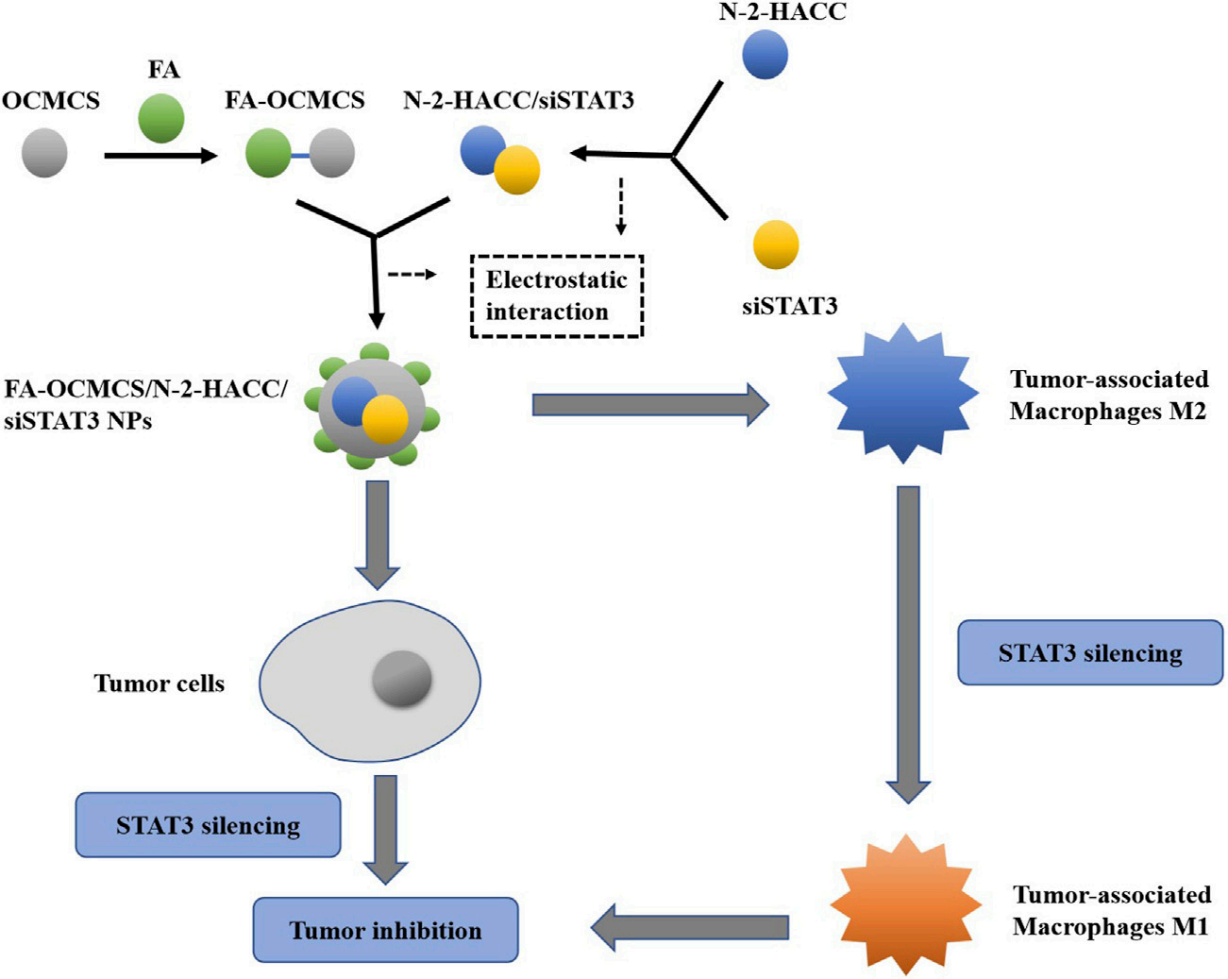
The construction of the NPs and dual-targeting pathways. With FA as the target ligand and OCMCS as the delivery carriers, NPs can be constructed by electrostatic interaction to achieve the targeted delivery of siSTAT3 to tumor-associated macrophages M2. By inhibiting the expression of STAT3, the NPs can transform M2 into M1 phenotype to inhibit tumor cells or directly deliver to tumor cells to induce apoptosis.

Chitosan (CS), a derivative of chitin, is a natural biopolymer with biocompatibility, biodegradability, nontoxicity, antibacterial activity, wound healing ability, and anticancer properties (Anitha et al., 2009). Because of these properties, it has many applications such as biomaterials for tissue engineering and wound healing, and as carriers for drug, gene, and polypeptides deliveries (Anitha et al., 2009). However, the poor solubility of CS in water limits its application. In order to overcome this defect, the functional groups of CS were modified by hydroxyl and amino groups to form CS derivatives such as carboxymethyl CS (CMC) which improved CS solubility by carboxymethylation as a hydrophilic modification that has numerous biomedical applications such as wound healing, bioimaging, tissue engineering, and drug/gene delivery (Mohammed et al., 2017; Shariatinia, 2018). Furthermore, CMC NPs have been obtained by ionic crosslinking of CMC with TPP or CaCl_2_ (Anitha et al., 2009). NPs prepared by CS and CS derivatives typically possess a positive surface charge and mucoadhesive properties which can adhere to the mucus membranes and release the drug payload in a sustained manner. CS-based NPs have various applications in nonparenteral drug delivery for the treatment of cancer. CS NPs also exhibit pH-dependent drug release because of the solubility of CS. CS derivatives alter the release of drugs from the NPs, afford tunable drug release, and impact the pharmacokinetic profile of the loaded drugs (Mohammed et al., 2017). To further improve delivery efficiency and cancer specificity, a strong emphasis has been placed on developing CS-based NPs with active tumor-targeting ability. Active targeting can be achieved by functionalizing CS and its derivatives with tumor-targeting ligands, such as folic acid (FA), antibodies, peptides, biotin, and avidin, which can recognize and bind to specific receptors that are unique to cancer cells (Prabaharan, 2015). As shown in Figure 5, FA-OCMCS can construct a small interfering RNA (siRNA) delivery system through electrostatic interaction along with N-2-HACC. N-2-HACC can effectively pack siRNA into FA-OCMCS which is suitable for cell internalization. Here, the dual-targeting system FA-OCMCS/N-2-HACC/siSTAT3 NPs prepared by electronic self-assembly is composed of FA-conjugated OCMCS and N-2-HACC/siSTAT3, targeting the overexpressed folate receptors on the surface of both LLC cells and M2 type macrophages. NPs induce cell apoptosis of tumor cells, and FA-OCMCS/N-2-HACC carriers protect siSTAT3 from serum degradation (Chen et al., 2020).

**FIGURE 6:**
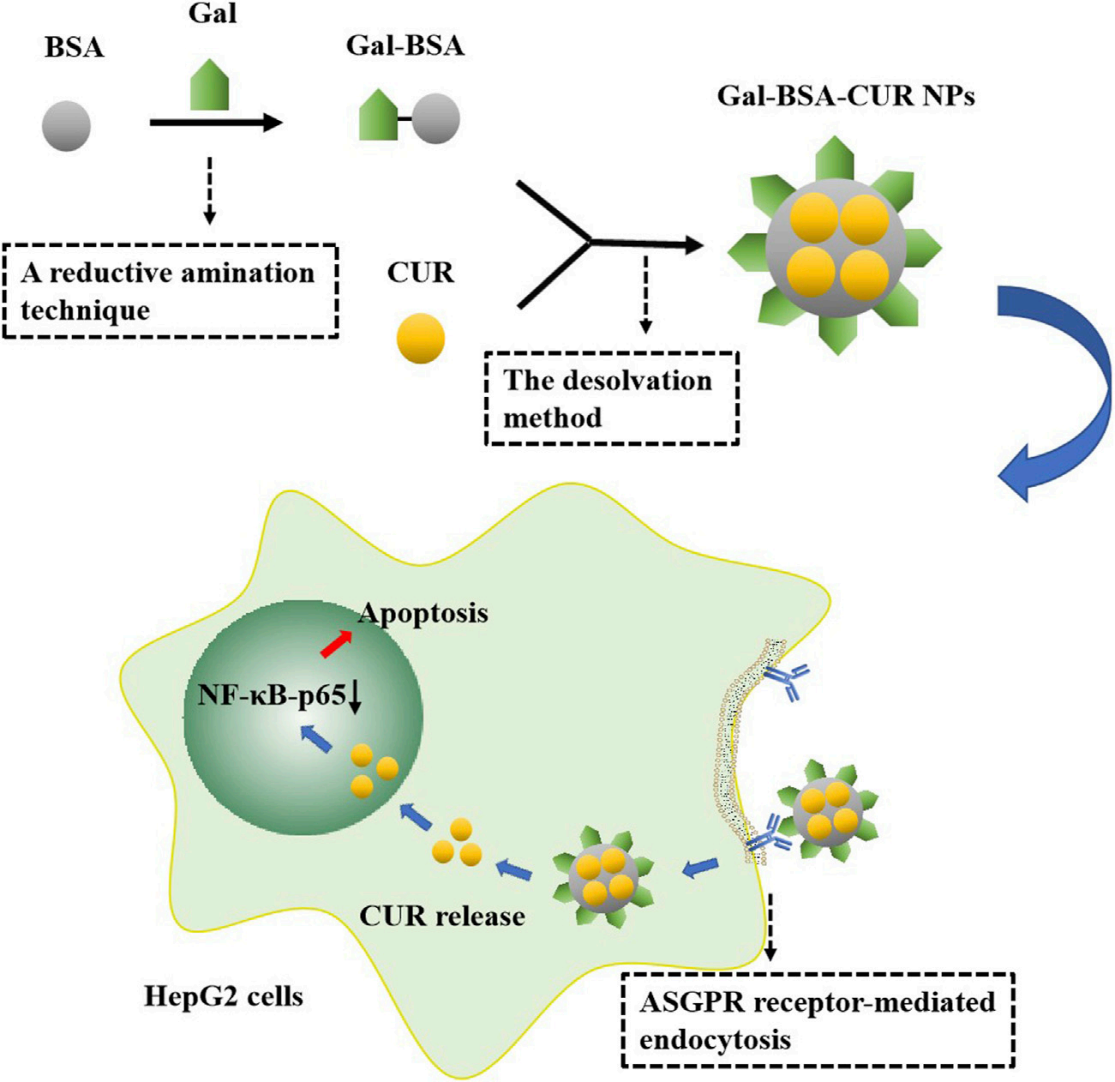
Synthesis of Gal-BSA-CUR NPs and antitumor effect. Gal-BSA was prepared by a reductive animation technique. Then, Gal-BSA-CUR NPs were synthesized by the desolvation method. The NPs taken up into cells through ASGPR receptor–mediated endocytosis releases CUR, which can inhibit the expression of NF-κB-p65 in the nucleus and induce cell apoptosis.

Albumin, the most abundant plasma protein synthesized in the liver, as an acidic and hydrophilic protein, plays an important role among protein-based nanomaterials as a carrier for targeted delivery of anticancer drugs which improves tumoricidal activity (Karimi et al., 2016; Wang and Zhang, 2018). Bovine serum albumin (BSA) is a globular non-glycosylated protein that consists of 583 amino acids, bound in a single chain with an approximate molecular weight of 69 kDa. BSA has widely been used in the preparation of nanomedicine due to its availability, low cost, purification, and stability (Lamichhane and Lee, 2020). BSA NPs, as versatile protein carriers for drug delivery, have been shown to be nontoxic, non-immunogenic, of low cost, biocompatible, easily metabolized *in vivo*, and soluble in water (Huang et al., 2018). In general, loading BSA NPs with antitumor drugs or therapeutic agents with different physicochemical properties can be achieved through two ways: 1) covalent conjugation: drugs or therapeutic agents such as curcumin (CUR) could be covalently conjugated with amino and carboxylic groups on BSA (Fu et al., 2016; Huang et al., 2018; Wang and Zhang, 2018); 2) non-covalent conjugation: non-covalent approaches, including encapsulation, hydrophobic interaction, coordination chemistry, and electrostatic interaction, can be performed on drugs or therapeutic agents such as DOX, 5-FU, PTX loading (Wang and Zhang, 2018). BSA surface engineering not only renders nanomaterials with hydrophilic and biocompatible properties but also provides active chemical groups for conjugating the targeted ligands, such as FA, monoclonal antibodies, and galactose (Huang et al., 2018; Lamichhane and Lee, 2020; Wang and Zhang, 2018). Figure 6 shows CUR-loaded galactosylated BSA NPs (Gal-BSA-CUR NPs) developed to improve CUR solubility, release effect, and bioavailability. The asialoglycoprotein receptor (ASGPR), as an important target molecule for high expression in liver cancer HepG2 cells, has a high affinity with Gal, which could significantly facilitate cell endocytosis of Gal-BSA-CUR NPs. As a targeted anticancer drug, the NPs can effectively inhibit HepG2 cell proliferation and cell migration and induce cell apoptosis. In addition, it has been proved that the pro-apoptotic effect of Gal-BSA-CUR NPs may be related to the inactivation of NF-κB-p65 (Huang et al., 2018).

**FIGURE 7:**
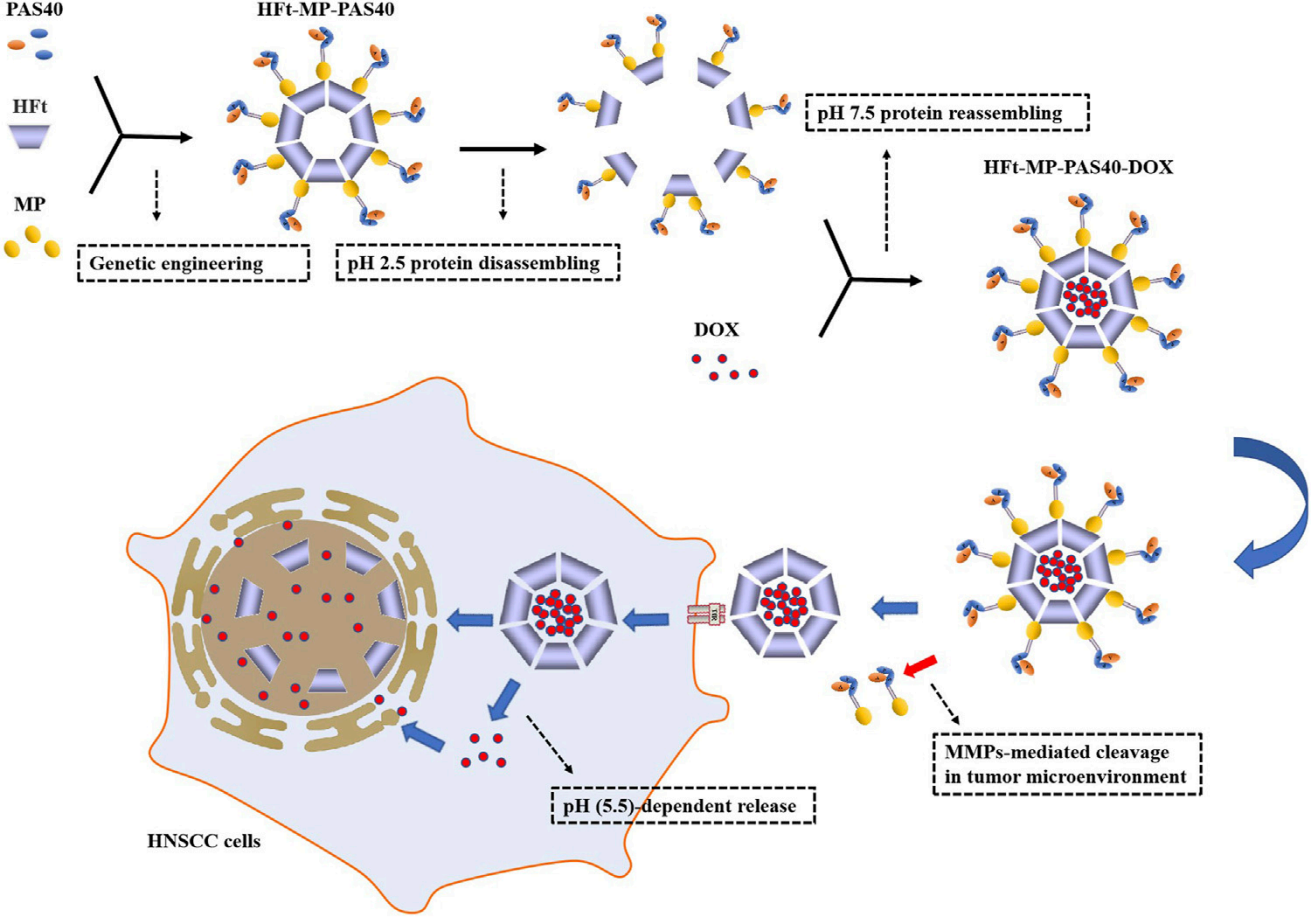
Assembly of HFt-based constructs, DOX encapsulation, and specific tumor suppression. PAS40: a polypeptide sequence of 40 residues rich in proline (P), alanine (A), and serine (S) residues; MP: a short motif sequence for responding to proteolytic cleavage by tumor matrix metalloproteases (MMPs). HFt-MP-PAS40 is assembled from PAS40, MP, and HFt through genetic engineering. The protein disassembling at pH 2.5 and then reassembling at pH 7.5 is to encapsulate DOX at the same time. PAS40 can hamper the interaction of DOX-loaded HFt with TfR1 on the surface of normal cells. MP between HFt subunit and the outer PAS polypeptide is processed by MMPs–mediated cleavage in the tumor microenvironment to remove PAS so that unmasked HFt can be specifically taken up by TfR1 overexpressed in tumor cells. In addition to a part of DOX that is pH (5.5)-dependent released/translocated in the cytoplasm and then diffuses to the nucleus, most of it is released by degradation of HFt in the nucleus, which ultimately leads to HNSCC damage.

Ferritin, a cage-like protein found in almost all living organisms, is made of 24 subunits arranged in octahedral 4-3-2 symmetry with an outer diameter of 12 nm and inner diameter of 8 nm, having its inner cavity connected to the outside by eight channels formed by the symmetrical positioning of its subunits on the shell, which allows the entry and exit of iron and other cations (Calisti et al., 2018). Ferritin tetraeicosamer could be reversibly disassembled at the extremely acidic pH of 2.5 or basic pH of 13.0, and then the protein could self-assemble into a properly folded protein nanocage at neutral pH (Palombarini et al., 2020). Based on these nanocage properties, ferritins have been efficiently loaded with drugs, fluorescent molecules, or contrast agents and used as drug delivery vectors and tools for bioimaging (Truffi et al., 2016). Ferritin has been reported to selectively target tumor cells that overexpress the Tf receptor TfR1 (CD71) (Calisti et al., 2018). Furthermore, mammalian ferritins include two highly conserved subunits: the heavy chain (H; 21 kDa) and light one (L; 19 kDa) (Palombarini et al., 2020). Human ferritin heavy chain (HFt) as carriers, efficiently delivering chemotherapeutics (DOX, CUR, or siRNA) to cancer cells, had been shown (Truffi et al., 2016). In Figure 7, HFt-MP-PAS40 is shown as a genetically engineered human ferritin heavy chain (HFt)–based construct that is able to efficiently encapsulate DOX, forming more stable complexes (HFt-MP-PAS40-DOX) with a longer half-life *in vivo*, which increases antitumor effects in comparison to the free drug in several squamous cell carcinomas (SCCs) of the head and neck (HNSCC) cell lines. The study also found HFt-MP-PAS40-DOX had less cardiotoxic and higher tolerated dose in terms of safety *in vivo*. High efficacy and small side effects have made it an extremely promising nanocarrier in clinical research and application for the treatment of tumors (Falvo et al., 2016; Fracasso et al., 2016; Damiani et al., 2017).

### Liposomes

**FIGURE 8:**
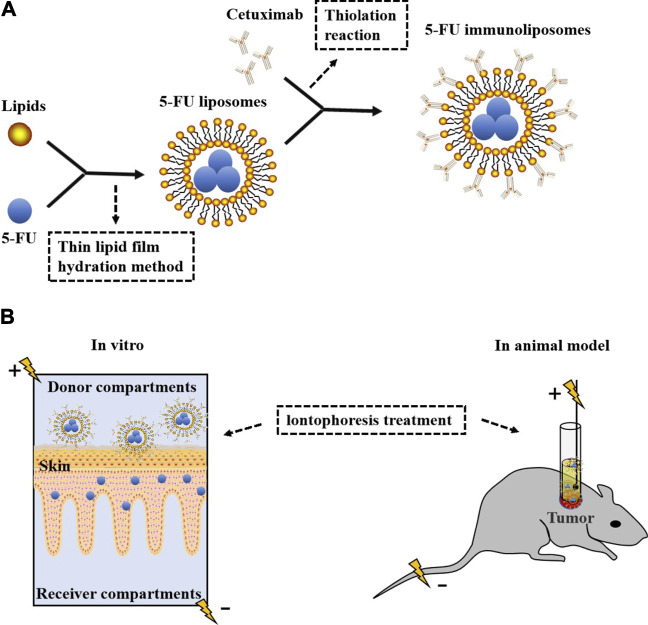
Preparation of ILs and iontophoresis treatment *in vitro* and *in vivo*. **(A)** 5-FU liposomes were prepared by coating 5-FU with lipids according to the drug-to-lipid ratio of 0.1 by the thin lipid film hydration method and then connected with thiolated cetuximab to form 5-FU ILs. **(B)** *In vitro*, iontophoresis with ILs increased the penetration rate of 5-FU into the skin and accumulated in the living epidermis but did not enter the receiver components. Immunoliposome ion delivery is more effective in treating tumors in animal models.

Liposomes are vesicles which can carry both hydrophobic and hydrophilic drugs, with one or more concentric phospholipid bilayers separated by aqueous compartments, and have been extensively studied for many years (Feng et al., 2017; Riaz et al., 2018). Liposomes being biologically inert and biocompatible have been used in the delivery of anticancer drugs which do not cause unwanted toxic or antigenic reactions (Bingham et al., 2010; Feng et al., 2017; Schwendener, 2007; Wang et al., 2005). And furthermore, liposomes can be modified with suitable ligands (peptides, antibodies, etc.) to form targeted liposomes and use overexpressed receptors as docking sites to deliver anticancer drugs (Riaz et al., 2018). In Figure 8, cetuximab-ILs enhance liposomal uptake by EGFR-positive cancer cells. An IL containing 5-FU modified by cetuximab has been developed, which can improve the efficacy of SCC by combining anti–EGFR antibody with a chemotherapeutic drug. Iontophoresis with ILs can increase the penetration of 5-FU into SCCs, and it is more effective than subcutaneous injection in reducing cell proliferation and invasion, which has become a more promising therapeutic strategy for SCC (Petrilli et al., 2018).

### Exosomes

**FIGURE 9:**
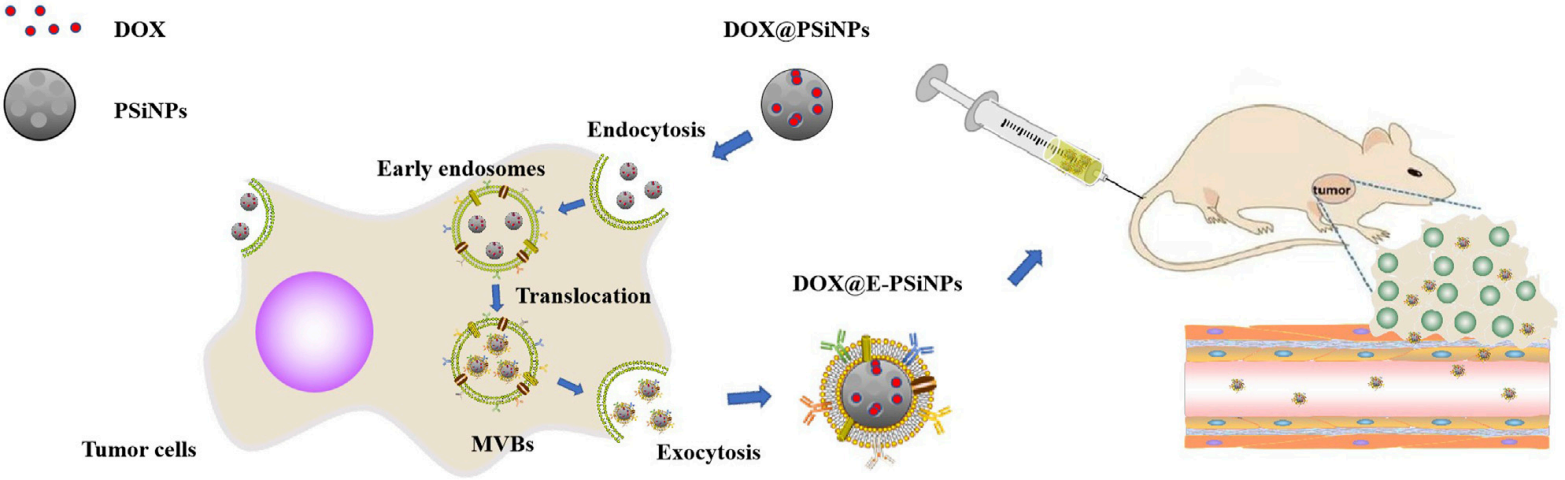
Preparation and efficacy of DOX@E-PSiNPs as tumor-targeted drugs. DOX@PSiNPs are taken up into tumor cells through endocytosis and localized in multivesicular bodies (MVBs) to prepare DOX@E-PSiNPs. With the fusion of MVBs and cell membrane, DOX@E-PSiNPs are released into the extracellular space through exocytosis. After intravenous injection, DOX@E-PSiNPs efficiently accumulate in tumor tissues and penetrate deeply into tumor parenchyma.

Exosomes are extracellular vesicles secreted by mammalian cells. They consist of a phospholipid bilayer cell membrane, are nanosized (50- to 100-nm) cup-shaped structures under the transmission electron microscope (Batrakova and Kim, 2015; Théry et al., 2002; van den Boorn et al., 2013). There are various labeled proteins and ligand proteins on the surface of exosomes, including ALIX, tetraspanins (CD9, CD63, CD81), integrins, and cell adhesion molecules (CAM) that reflect their intracellular endosomal origin, which attach to and deliver their payload to target cells (Batrakova and Kim, 2015; van den Boorn et al., 2013). Exosomes have stability, biocompatibility, low immunogenicity, and low toxicity in circulation and can be used to target disease tissues and/or organs based on their properties and origin with specific cellular propensity (Batrakova and Kim, 2015). Recently, exosome–biomimetic NPs, constructed by exosomes as natural biomaterials and functionalized NPs, have attracted much attention as an effective drug delivery platform. Generally, exosome–biomimetic NPs are fused by iterative physical extrusion or freeze/thaw cycles, which might affect the protein integrity on exosome membranes, interfering with cancer therapy. Here, luminescent PSiNPs have been used to synthesize a biocompatible tumor cell–exocytosed exosome biomimetic PSiNPs (E-PSiNPs) as a drug carrier for targeted cancer chemotherapy owing to their excellent drug-loading capacity, high biocompatibility, and biodegradability. Exosome-sheathed DOX-loaded PSiNPs (DOX@E-PSiNPs) can be used as a carrier for DOX-targeted delivery. *In vitro*, DOX@E-PSiNPs can induce low expression of multidrug-resistant protein P-glycoprotein (P-gp), resulting in decreased cell membrane fluidity, which enhances intracellular retention, and its targeting ability to tumor cells is regulated by CD54 (ICAM1), making these exhibit strong cellular uptake. Compared with free DOX or DOX@PSiNPs, DOX@E-PSiNPs have a more excellent cytotoxicity against cancer stem cells (CSCs). *In vivo*, following intravenous injection, DOX@E-PSiNPs display enhanced tumor accumulation, tumor penetration, and cross-reactive cellular uptake by bulk cancer cells and CSCs, resulting in augmented DOX enrichment in total tumor cells and side population cells, which can further improve anticancer efficacy (Figure 9) (Yong et al., 2019).

### Dendrimer

**FIGURE 10:**
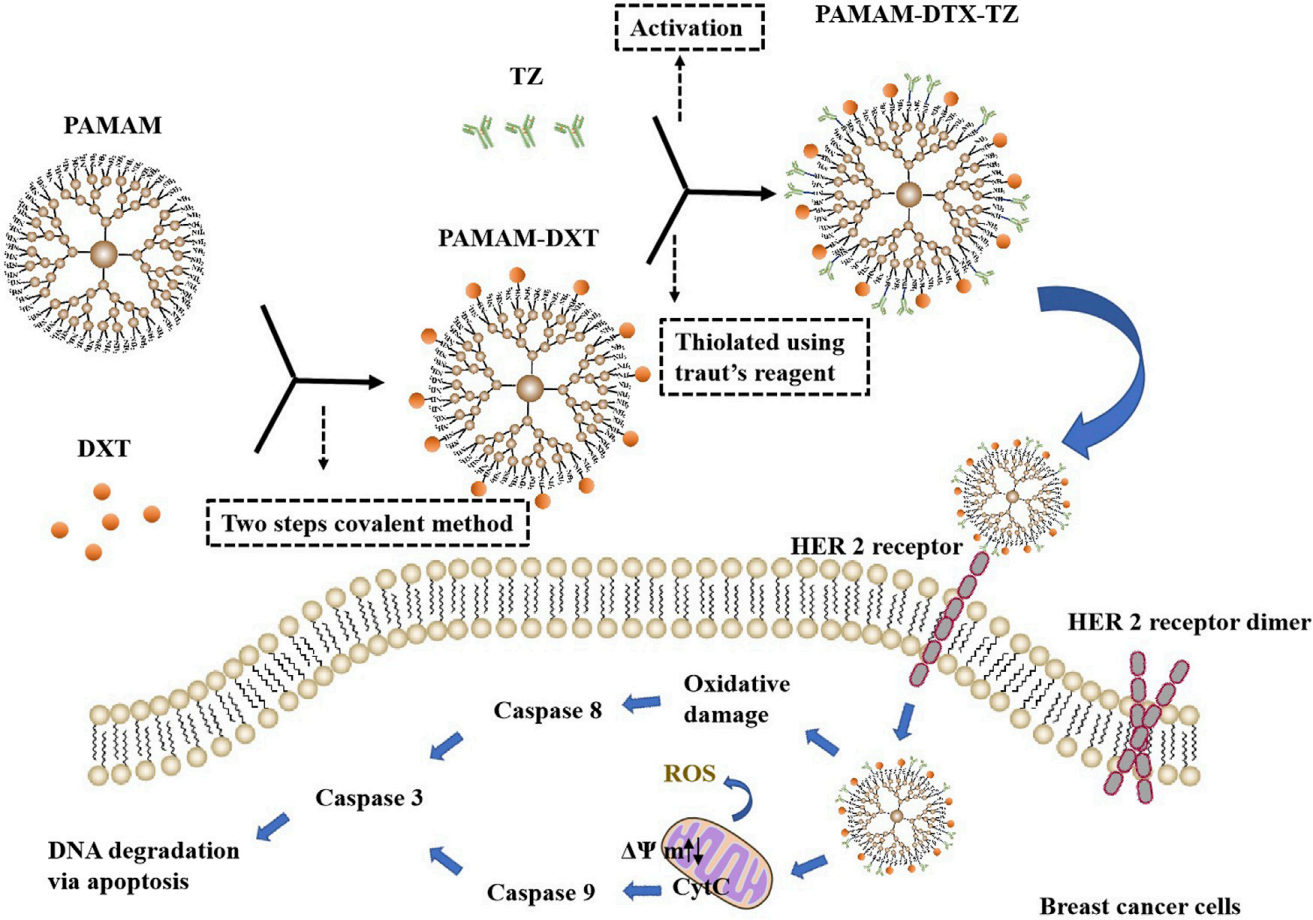
The synthesis of PAMAM-DTX-TZ and three main modes of action. The linking of DTX to PAMAM dendrimers was done using a two-steps covalent method. Then, PAMAM-DTX thiolated using Traut's reagent reacted with activated TZ to synthesize PAMAM-DTX-TZ. PAMAM-DTX-TZ showed the different potential mechanisms of cytotoxic action for breast cancer cells. The mechanism is not only associated with the blocking of the HER 2 receptor but also achieved through ROS generation (oxidative damage → caspases 8 → caspases 3 → DNA degradation via apoptosis) or changes in mitochondrial membrane potential (mitochondria damage → CytC → caspases 9 → caspases 3 → DNA degradation via apoptosis).

Donald A Tomalia succeeded in making the first poly (amidoamine) (PAMAM) dendrimers in 1979 and first published his seminal work in 1985 (Chauhan, 2018; Tomalia, 2012). Dendrimers are a relatively new type of synthetic dendritic polymers with three-dimensional, branched, highly monodispersed, stepwise synthetic macromolecular nanoscopic (1–100 nm) architecture (Singh et al., 2008; Zhang et al., 2018). Dendrimer architecture offers a new strategy for solubilizing water-insoluble drugs and has three main sites for drug entrapment by using various mechanisms, including void spaces (by molecular entrapment), branching points (by hydrogen bonding), and outside surface groups (by charge–charge interactions), which provides a unique nanocontainer property for drug delivery (Chauhan, 2015, Chauhan, 2018). It also provides a polyvalent platform to bind multiple biological targets improving therapeutic effects, which have been used to produce a novel nanodrug for antiviral and anti-inflammatory treatments (Chauhan, 2015). Dendrimers have been reported to increase transdermal permeation and specific drug targeting (Chauhan, 2018). As a fascinating delivery method for potential anti-inflammatory treatment strategies, dendrimers have successfully delivered indomethacin via transdermal routes as a transdermal permeation enhancer (Chauhan, 2018; Singh et al., 2008). However, in terms of cancer treatment, dendrimers-based targeted delivery has been the most widely used indispensable tool and has had high biocompatibility with low side effects on normal cells, immune function, and blood components, and enhanced solubility, stability, and oral bioavailability of various antitumor drugs (Chauhan, 2015, Chauhan, 2018). Trastuzumab (TZ)-grafted dendrimers were synthesized to achieve effective delivery of DTX to human epidermal growth factor receptor 2 (HER2)–positive breast cancer cells and which were more selective and had higher antiproliferation activity than HER2-negative cells. Compared with unconjugated dendrimers, TZ-conjugated dendrimers displayed stronger targeting and higher cellular internalization, and also lower hemolytic toxicity and longer circulation half-life. Furthermore, the level of ROS, the mitochondrial membrane potential, cell cycle distribution, and the activity of caspases 3/7, 8, and 9 of PAMAM-DTX-TZ conjugates were determined and compared with free DTX. These findings could help to develop a better therapeutic profile with HER2-positive breast cancer for DTX (Figure 10) (Kulhari et al., 2016; Marcinkowska et al., 2019a; Marcinkowska et al., 2019b).

## The Antitumor Effect and Mechanism of Different Antitumor Drugs in Delivery Carriers

At present, different kinds of antitumor drugs, such as DOX, PTX, DTX, CUR, and siRNA, combined with a variety of delivery carriers have been approved for clinical treatment (Wicki et al., 2015). Other antitumor drugs such as metformin (MET) and 5-fluorouracil (5-FU) have been widely studied in the treatment of cancer with drug delivery systems, which have potential clinical application value (Li et al., 2008; Aydın et al., 2020). As shown in Table 1, a large number of studies have been carried out on the treatment of various cancers by these antitumor drugs through various delivery carriers, which provide valuable experience for more long-term effective clinical cancer treatment in the future.

**TABLE 1:** Different anticancer drugs, chemical structures, delivery carrier, and types of cancer.

Drug	Chemical structures	Delivery carrier	Type of cancer	References
DOX	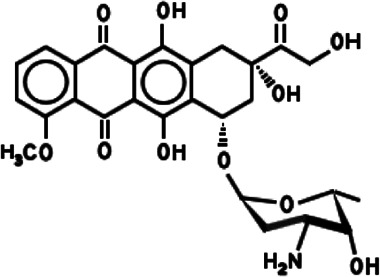	CMC NPs	Breast and lung cancers	Guo et al. (2005); Shi et al. (2006); Wang et al. (2009); Cho et al. (2010); Shen et al. (2012); Slingerland et al. (2012); Su et al. (2014b); Rivankar (2014); Damiani et al. (2017); Ji et al. (2018); Li et al. (2018); Xia et al. (2018); Zhang et al. (2018); Lorusso et al. (2019); Li et al. (2020a); Nie et al. (2020); Li et al. (2021)
		BSA NPs	Liver cancer	
		HFt NPs	Colon cancer	
		Liposomes	Cervical cancer	
		Exosomes	Ovarian carcinoma	
		Dendrimer	Glioma	
		PSiNPs	HIV-associated Kaposi's sarcoma	
		AuNPs		
		AgNPs		
PTX and DTX	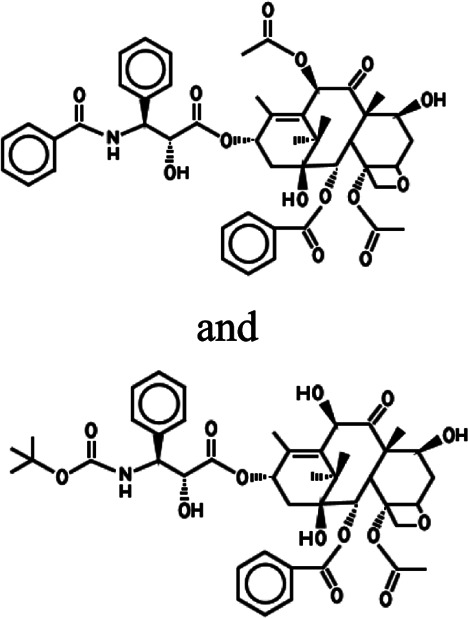	CMC NPs	Breast cancer	Shi et al. (2012b); Maya et al. (2013); Liu et al. (2014); Qu et al. (2014); Yang et al. (2014); Xu et al. (2017); Marcinkowska et al. (2019a); Ma et al. (2021)
		HFt NPs	lung cancers	
		Liposomes	liver cancer	
		Dendrimer	laryngeal cancer	
		GO	melanoma	
			glioblastoma	
			skin squamous cell carcinoma	
CUR	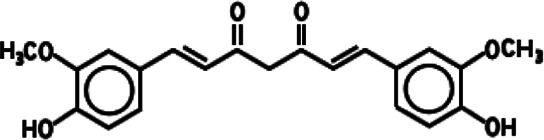	CMC NPs	Breast and lung cancers	Mach et al. (2009); Shi et al. (2012a); Anitha et al. (2012b); Manju and Sreenivasan (2012); Ranjan et al. (2013); Anitha et al. (2014); Hatamie et al. (2015); Feng et al. (2017); Jose et al. (2018); Malekmohammadi et al. (2018); Rahimi Moghaddam et al. (2019); Wang et al. (2019)
		BSA NPs	Liver cancer	
		Liposomes	Pancreatic cancer	
		Exosome	Colon cancer	
		GO	Cervical cancer	
		AuNPs	Skin cancer	
			Prostate cancer	
			Colorectal cancer	
			Glioma	
			Osteosarcoma	
siRNA		CMC NPs	Breast and lung cancers	Greco et al. (2016); Yang et al. (2016); Chen et al. (2017); Kamerkar et al. (2017); Haghiralsadat et al. (2018); Mendt et al. (2018); Sun et al. (2018); Rakhit et al. (2019)
		PSiNPs	Liver cancer	Mitra et al. (2013); Xie et al. (2014); Yue et al. (2017); Tong et al. (2018); Luan et al. (2019); Qu et al. (2019); Bai et al. (2020); Pędziwiatr-Werbicka et al. (2020); Tang et al. (2020); Tao et al. (2020); Yan et al. (2020)
		GO	Pancreatic cancer	
		AgNPs	Colon cancer	
		AuNPs	Cervical cancer	
		Liposomes	Prostate cancer	
		Exosomes	Colorectal cancer	
		Dendrimer	Bladder cancer	
			Glioma	
			Osteosarcoma	
			Glioblastoma	
			Retinoblastoma	
MET	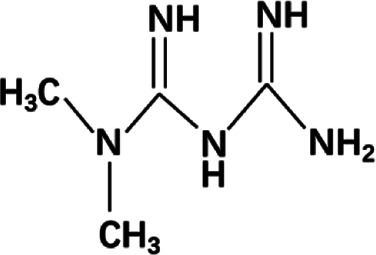	CMC NPs	Breast and lung cancers	Snima et al. (2012); Snima et al. (2014); Jose et al. (2015); Shi et al. (2017); De et al. (2019); Lee et al. (2019); Shukla et al. (2019); Lu et al. (2020); Yang et al. (2020); Xiong et al. (2021)
		BSA NPs	Liver cancer	
		Liposomes	Pancreatic cancer	
		GO	Gastric cancer	
		AuNPs		
5-FU	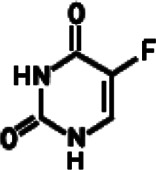	CMC NPs	Breast and lung cancers	Anitha et al. (2012a); Anitha et al. (2014); Su et al. (2014a); Ganeshkumar et al. (2014); Udofot et al. (2015); Ngernyuang et al. (2016); Mani et al. (2018); Nawaz and Wong (2018); Alomrani et al. (2019); Chinnaiyan et al. (2019); Gajendiran et al. (2019); Liszbinski et al. (2020); Mulens-Arias et al. (2021)
		BSA NPs	Pancreatic cancer	
		AuNPs	Colon cancer	
		AgNPs	Cervical cancer	
		Liposomes	Colorectal cancer	
		Dendrimer	Melanoma	
			Cholangiocarcinoma	

### DOX

DOX, also known as adriamycin, is an anthracycline antibiotic isolated from the *Streptomyces peucetius* spp possessing aglyconic and sugar moieties (Carvalho et al., 2009; Chen et al., 2018). The aglycone consists of a tetracyclic ring with adjacent quinone–hydroquinone groups, a methoxy substituent, and a short side chain with a carbonyl group. The sugar, called daunosamine, is attached by a glycosidic bond to one of the rings and consists of a 3-amino-2,3,6-trideoxy L-fucosyl moiety (Carvalho et al., 2009). DOX induces cell death or cell growth arrest through a variety of molecular mechanisms, including inhibition of topoisomerase II, intercalation of DNA, and production of free radicals. Because of its broad-spectrum antitumor effect and low price, DOX has been widely used for the treatment of many types of cancers (Chen et al., 2018). However, DOX has antitumor activity and cardiotoxicity, which limits its clinical application (Rivankar, 2014). DOX carrier has been developed as another promising alternative method and can reduce the frequency of drug delivery to reduce side effects by controlling the drug release. Pegylated liposomal DOX (PLD) has been used in clinics as an antitumor drug, which has lower toxicity and better tolerance to a variety of tumors, including HIV-associated Kaposi's sarcoma, ovarian carcinoma, breast cancer, and hematological malignancies (Slingerland et al., 2012; Rivankar, 2014). PLD, whether used alone or in combination with other drugs, is an option for palliative diseases (e.g., ovarian cancer) and is a safe and effective alternative to anthracycline or other antitumor drugs (e.g., platinum). PLD is an enhancement of the traditional DOX formulation, showing reduced cardiotoxicity and improved pharmacokinetic properties (Lorusso et al., 2019). Based on PSiNPs conjugated with IR820 dye and DOX molecule, the combination treatments including chemotherapy and PTT could offer a synergistic effect to destruct drug-resistant cancer cells with a higher therapeutic efficacy. DOX and photothermal agents (IR820 dye) have been successively incorporated into amine-terminated PSiNPs (NH2-PSiNPs) via electrostatic attractions, to prepare DOX/IR820/NH2-PSiNPs nanocomposites with a high loading amount of DOX (13.3%, w/w) and IR820 (18.6%, w/w), respectively. Dual pH/NIR light triggering the release of DOX molecules was found in DOX/IR820/NH2 PSiNPs, which has been helpful in delivering DOX molecules into drug-resistant cancer cells and then improving their intracellular release and accumulation located in the cellular nuclei (Xia et al., 2018). Some studies have also evaluated the potential of CMC NPs as DOX delivery carriers, in which the molecular weight (MW) and degree of substitution (DS) of CMC directly affect the formation of NPs and drug release, and CMC with high MW and DS can improve the encapsulation efficiency of DOX and hinder the release of DOX (Shi et al., 2006). Based on the optimization strategy of DOX delivery carrier, a novel FA-conjugated CMC ferroferric oxide (Fe_3_O_4_)–doped cadmium telluride quantum dot (CdTe QD) NPs (CFLM NPs) was developed, which has a high drug-loading rate, low cytotoxicity, and good cytocompatibility, in addition to an encapsulated DOX to realize targeted delivery and cell imaging (Shen et al., 2012). CFLM NPs are transported into HepG2 cells through FA receptor–mediated endocytosis mechanism, and they have a strong intense superparamagnetic effect and photoluminescence (PL) property due to Fe_3_O_4_ and CdTe QD. The magnetic delivery carrier labeled fluorescent dye could be tracked to obtain images of migration and anchoring of DOX *in vivo*. The outer CMCH shell with good functionality provides not only protection from CdTe QD escape and fluorescent quenching but also a potent platform for modifying FA and DOX binding (Guo et al., 2005; Guo et al., 2006; Wang et al., 2009; Cho et al., 2010; Shen et al., 2012). The development and application of multifunctional DOX-conjugated PAMAM dendrimers as a promising nanodevice for pH-responsive drug release and targeted cancer chemotherapy has been widely reported (Kaminskas et al., 2011; Zhang et al., 2018). Generation 5 (G5) of PAMAM dendrimers as the core carrier for DOX has allowed functionalization of the surface with compounds such as N-acetylgalactosamine (NAcGal) via an pH-sensitive cis-aconitic (CA) linkage which escapes recognition by healthy hepatocytes and liver macrophages by targeting the asialoglycoprotein receptor (ASGPR) overexpressed on hepatic cancer cells (Kuruvilla et al., 2017). The G5 NHAc-FA-DOX conjugates are formed with covalently conjugated DOX onto the periphery of partially acetylated and FA-modified G5 PAMAM dendrimers through pH-sensitive cis-aconityl linkage to form that which are capable of specifically targeting cancer cells via FAR-mediated endocytosis, and exhibiting a significantly enhanced therapeutic efficacy (Zhang et al., 2018). A study showed ^131^I-antiAFPMcAb-DOX-BSA-NPs have been successfully prepared with good monodispersion and high radiochemical purity and confirmed the preferably combinatorial therapeutic efficacy on hepatoma which could significantly inhibit the hepatoma tumor growth with the strategy of combinatorial radioimmunotherapy and chemotherapy (Ji et al., 2018). Furthermore, based on BSA NPs loading DOX and being modified with both lactoferrin (Lf) and mPEG2000, a dual-targeting drug delivery system was designed which could target the primary brain capillary endothelial cells (BCECs) and glioma cells (C6) and improve drug release efficiency, BBB penetration, and targeted antitumor properties (Su Z. et al., 2014). In addition, as a promising drug carrier with low immunogenicity, high biocompatibility, and delivery efficiency, exosomes isolated from A33-positive LIM1215 cells (A33-Exo) could be also used to load DOX. To target A33-positive colon cancer cells, the A33Ab-US-Exo/DOX complex was designed via A33-Exo/DOX combining with A33 antibodies-coated surface-carboxyl superparamagnetic iron oxide NPs (US) (A33Ab-US). A33Ab-US-Exo/DOX has excellent targeted antitumor ability and prolongs survival in mice with reduced cardiotoxicity (Li et al., 2018).

### PTX and DTX

PTX is a tricyclic diterpenoid compound naturally produced in the bark and needles of *Taxus brevifolia* (Zhu and Chen, 2019). PTX, as a mitotic inhibitor, promotes tubulin polymerization and blocks the progression of mitosis which blocks cells in G2 and M phases of the cell cycle resulting in cell death (Yardley, 2013; Wei et al., 2017; Zhu and Chen, 2019). Because of its unique anticancer mechanism, PTX is widely used for the treatment of various cancers, including cervical, breast, ovarian, brain, bladder, prostate, liver, and lung cancers (Wei et al., 2017; Zhu and Chen, 2019). PTX is not only isolated from plants with low content and at a high price but is also complex in structure and difficult to synthesize (Nicoletti et al., 1994; de Weger et al., 2014; Zhu and Chen, 2019). Therefore, it is urgent to develop a new kind of taxanes. As a semisynthetic product of PTX, DTX is produced by esterification of 10-deacetylbaccatin III, which is isolated from the needles of the European yew tree *Taxus baccata*, providing a renewable source of natural products (Pazdur et al., 1993; de Weger et al., 2014). Moreover, DTX can promote tubulin polymerization better than PTX and has higher cytotoxicity. It has shown good therapeutic effect on breast, ovarian, and non–small-cell lung cancers in clinical trials (Nicoletti et al., 1994). Although taxanes can improve the survival rate, they are limited in their clinical treatment of cancer due to their low water solubility, poor selectivity, drug resistance, and side effects (Yardley, 2013; Wei et al., 2017). In order to improve these characteristics, it is one of the most feasible strategies to modify PTX and DTX to construct an excellent drug delivery system. O-CMC NPs, as the delivery carrier of drug delivery systems, are conjugated with cetuximab (Cet) on the surface. They are used to deliver PXL to overexpressed EGFR cancer cells (A549, A431, and SKBR3) and show good sustained release and antitumor effects (Maya et al., 2013). Similarly, the O-CMC NPs modified by glycyrrhizin (GL) could specifically deliver PTX into hepatocellular carcinoma (HCC), which results in remarkably enhanced cytotoxicity *in vitro* and antitumor efficacy *in vivo* compared to PTX injection and unmodified O-CMC NPs (Shi L. et al., 2012). As the optimization strategy of targeting a single cell line, a double-targeted drug delivery system has been proposed. Here, based on the modification of cyclic NGR peptides (cNGR) to DXT carrier, the double-targeted DTX-CMCS-PEG-NGR (DTX-CPN) conjugates were successfully synthesized that could be targeted to CD13 overexpressed HUVEC cells and B16 cells and achieved a novel double targeting cancer therapy in the inhibition of tumor growth and new angiogenesis (Liu et al., 2014). The combination therapy of double-drug delivery overcame the limitation of single-drug delivery and has become the standard treatment in clinics (Qu et al., 2014). A study had developed liposome-encapsulated DTX and siRNA against the ABCG2 gene (ABCG2-siRNA) which were used in combination therapy and could be targeted to the EGFR receptor on the surface of Hep-2 laryngeal cancer cells by conjugating GE11 peptide, which had significantly enhanced apoptotic effects and antitumor activities (Xu et al., 2017). In particular, liposomes loading vascular endothelial growth factor (VEGF) siRNA and DTX with combination therapy and modified by the dual peptides (angiopep-2 and tLyP-1), specifically attaching two receptors (low-density lipoprotein receptor–related protein receptor and neuropilin-1 receptor) for brain tumor targeting and tumor penetration, were designed to show more superiority in antitumor efficacy with anti-angiogenesis and apoptosis effects compared with non-modified and single peptide–modified or single drug–loading liposomes, which provided a strategy with the dual peptides–guided combination therapy in inhibition of human glioblastoma cells (U87 MG) (Yang et al., 2014). In addition, the dual-targeting NP delivery system tLyP-1-HFt-PTX NPs was also developed which not only improved tumor penetration through an NRP-1–dependent internalization pathway but also bound to tumor cells by interacting with TfR1. Due to the dual receptor–mediated endocytosis process, tLyP-1-HFt-PTX NPs displayed stronger intracellular uptake efficiency and anti-invasion ability compared with HFt-PTX NPs and PTX. The BALB/c mice model also showed that tLyP-1-HFt-PTX had lower systemic toxicity and higher therapeutic efficacy *in vivo* (Ma et al., 2021).

### CUR

CUR is a polyphenolic compound extracted from the rhizomes of *Curcuma longa*, which is safe, nontoxic, antioxidant, antibacterial, anti-inflammatory, and anticancer. CUR has been widely used in the prevention and treatment of various cancers, including colorectal, gastric, breast, liver, esophageal, prostate, lung, and brain cancers, and leukemia in preclinical studies (Wei et al., 2018). However, due to its poor water solubility and low bioavailability, its pharmacological action at appropriate concentrations is limited. NPs as delivery carriers of CUR can improve internalization efficiency to reduce the required therapeutic dosages and toxicity and enhance the bioavailability (Huang et al., 2018). Anitha developed a cancer drug delivery of CUR-loaded N, O-CMC NPs and proved its validity. After that, Anitha further designed combinatorial N, O-CMC NPs based on 5-FU and CUR to enhance the antitumor effect, which excellently improved plasma concentrations under *in vivo* conditions (Anitha et al., 2012b; Anitha et al., 2014). Hatamie et al. (2015) described green reduction and functionalization of GO by CUR resulting in the π–π attachment of the CUR molecules onto reduced GO (rGO) sheets. Similarly, Malekmohammadi et al. (2018) prepared a new sandwich-like nanocomposite as a multifunctional smart nanocarrier for CUR-targeted delivery and cell imaging by immobilization of AuNPs on FA-modified dendritic mesoporous silica-coated rGO nanosheets (AuNPs@GFMS). A low pH value and NIR laser irradiation can stimulate the release of CUR by causing a decrease in the electrostatic and hydrophobic interactions between CUR and the nanocarrier, and CUR could further affect human breast cancer cells. It is noteworthy that AuNPs can act as good PTT agents for different cancer treatments. Hence, AuNPs can also be used as the carrier of CUR to improve therapeutic effects in cancer by PTT (Rahimi Moghaddam et al., 2019). Besides NPs, the combination of CUR and liposomes was also used to enhance the stability, bioavailability, targeting property, and antitumor efficacy of CUR (Feng et al., 2017). Mach et al. (2009) developed liposomal formulation of CUR (lipo-CUR) for the first time and determined minimum effective dose (20 mg/kg) and optimal dosing schedule (once daily, three times per week) for lipo-CUR in a xenograft human pancreatic cancer model. Ranjan et al. (2013) further verified the inhibition of lipo-CUR in tumor growth and angiogenesis in a human pancreatic tumor xenograft model. Other studies have shown that lipo-CUR could also effectively mitigate radiation pneumonitis, reduce fibrosis of the lung, and sensitize murine lung carcinoma LL/2 cells to irradiation (Shi HS. et al., 2012). Jose et al. (2018) showed effective skin cancer treatment by the iontophoretic (0.47 mA/cm^2^) method, by topical co-delivery of CUR and STAT3 siRNA using cationic liposomes made up of DOTAP and DOPE. Dual-functional liposomes for CUR and silk fibroin (SF) hydrogel were reported, and the feasibility of the CUR-SF hydrogel as a sealant administered after tumor resection was assessed (Laomeephol et al., 2020). Since hydrogel could excellently be used for tumor recurrence and metastasis prevention as a thermosensitive filler with *in situ* injectable performance, liposomal hydrogels (CSSH/CUR-lipo gel) formed via CUR-lipo coated with thiolated CS (CSSH) could be a promising novel drug delivery system which effectively delayed the release of CUR and exerted an excellent antitumor effect *in vitro* and *in vivo* (Li R. et al., 2020). In the targeted delivery system, RGD peptide or FA-modified CUR-lipo had been developed and proved to be an optimized carrier for targeting antitumor drug based on CUR (Wang et al., 2019; Mahmoudi et al., 2021). Finally, CUR could increase exosomal transcription factor 21 (TCF21) to suppress exosome-induced lung cancer (Wu et al., 2016) and upregulate exosomal miR-21 affecting angiogenic phenotype (Taverna et al., 2016). CUR could also reverse breast tumor exosomes-mediated immune suppression of NK cell tumor cytotoxicity (Zhang et al., 2007).

### siRNA

RNA interference (RNAi), as an important cancer treatment technology, can specifically and selectively knockdown the expression of target genes to silence the function of oncogenes (Rao et al., 2009; Lee et al., 2016; Xiao et al., 2017). siRNA as a cancer treatment can mediate the RNAi effect by suppressing the expression of the carcinogenic genes via selectively targeting the mRNA. Its drug delivery system has been designed to achieve the best anticancer effect (Lee et al., 2016; Singh A. et al., 2018). Using systemically delivered siRNAs, KRAS as a target of KRAS siRNA was silenced which regulated cellular proliferation and survival in lung and colon adenocarcinoma treatment (Pecot et al., 2014; Ying et al., 2016). Chen et al. (2017) developed novel redox-sensitive oligopeptide liposomes for co-delivery of PTX and survivin siRNA, which could inhibit PTX-induced upregulation of survivin expression, and further recovered the sensitivity of the 4T1 breast cancer cells to PTX. Dual-modified cationic liposomes by angiopep-2 and RNA Apt A15 also loaded with PXT and survivin siRNA could bind highly expressed low-density lipoprotein receptor–related protein (LRP) on the surface of the BBB and track CD133^+^ glioma stem cells, which effectively promoted apoptosis induced by co-delivery of PTX and survivin siRNA (Sun et al., 2018). Yang et al. (2016) described a novel siRNA targeting system that combined features of thermal and magnetic dual-responsive liposomes with cell-permeable peptides (CPPs)–siRNA conjugates (siRNA-CPPs). Magnetic fluid Fe_3_O_4_ could be used to thermally trigger siRNA-CPPs release, and siRNA-CPPs had a much stronger cell penetration ability than siRNA. However, due to the instability of liposomes, PEGylated liposomes were developed to improve *in vivo* stability of siRNA (Haghiralsadat et al., 2018). In addition, compared to liposomes, exosomes exhibit a superior ability to deliver RNAi and suppress tumor growth. Engineered exosomes could carry oncogenic KRASG12D siRNA or shRNA into human pancreatic orthotopic tumors that suppress KrasG12D expression, inhibit advanced metastatic disease, and increase overall survival (Kamerkar et al., 2017; Mendt et al., 2018; Rakhit et al., 2019). Exosomes isolated from human embryonic kidney 293 cells (HEK293) and mesenchymal stem cells that transport PLK-1 siRNA to bladder cancer cells *in vitro* and those isolated from bovine milk that deliver bcl-2 siRNA to human pancreatic cancer Panc28 and human colorectal cancer HCT-116 cells result in selective gene silencing, both of which have been reported (Greco et al., 2016; Tao et al., 2020). Targeting peptide tLyp-1-–modified exosomes by tLyp-1-lamp2b plasmids–loading SOX2 siRNA isolated from HEK293 cells have been proved to have high transfection efficiency into lung cancer and cancer stem cells which were able to knockdown the target gene SOX2 of cancer cells and reduce the stemness of cancer stem cells (Bai et al., 2020). CMC, as a carrier, functionalized with some groups such as guanidine group or polyethylene glycol (PEG) to optimize biofunctions, can deliver siRNA functionalized with polymer such as fluorescein isothiocyanate–CS hydrochloride or poly-β-amino ester to cancer cells (Xie et al., 2014; Tang et al., 2020; Yan et al., 2020). As a feature of optimizing biofunctions, PSiNPs with polyethyleneimine (PEI) capping were designed to achieve high-capacity loading of siRNA (92 µg of siRNA/mg PEI-PSiNPs) and optimize release profile (70% released between 24 and 48 h) (Tong et al., 2018). Structurally flexible triethanolamine (TEA) core PAMAM dendrimers, as stable NPs protecting siRNA from enzymatic degradation, were able to deliver Hsp27 siRNA effectively into prostate cancer (PC-3) cells and enhance cellular uptake of siRNA. Silencing of the hsp27 gene could induce caspase3/7–dependent apoptosis and inhibit PC-3 cell growth *in vitro* (Liu et al., 2009). It is noteworthy that glycyrrhetinic acid (GA) as a targeting ligand functionalized PEG–polyamidoamine dendrimer (dendrimer)–nano-GO conjugate efficiently delivered VEGFa siRNA into liver tumor tissues, eventually inhibiting the growth of the tumor tissue with enhanced targeting capability and no obvious pathological changes, which showed great promise in being used as an excellent nanocarrier in achieving clinical benefits (Qu et al., 2019). Based on the high stability of MtNPs, among them, AgNPs can be further modified by carbosilane dendrons as carriers of anticancer Bcl-xl siRNA which have surface activity. A marked increase in the hydrodynamic diameter of complexes accompanied by higher PDI values suggests the formation of AgNPs-Bcl-xl siRNA. Complexation with AgNPs provides protection of Bcl-xl siRNA from the action of RNase (Pędziwiatr-Werbicka et al., 2020). Yue et al. (2017) prepared various formulations of siRNA-conjugated AuNPs based on cores of 13-nm spheres, 50-nm spheres, and 40-nm stars. The size of the AuNP cores can influence cellular uptake and intracellular distribution of siRNA. PEI or PEG can usually be used to cap AuNPs and form stable complexes with siRNA (Mitra et al., 2013; Luan et al., 2019). The co-delivery complexes of EGFP siRNA and DOX were synthesized from the electrostatic interaction between positively charged EGFP siRNA-AuNPs-PEI and negatively charged poly-(lactic-co-glycolic acid)-DOX which resulted in a substantial downregulation of EGFP expression and intracellular accumulation of DOX (Kumar et al., 2017). At last, Guo et al. (2016) synthesized two novel AuNPs, namely AuNPs-PEG-Tf and AuNPs-PEI-FA, to enhance cell-specific uptake, with the purpose of providing efficiency for the siRNA delivery systems in the treatment of prostate cancer.

### MET

MET, a biguanide drug, has a great effect in improving the prognosis of cancer patients and preventing the occurrence of tumors. In the past decades, some epidemiological studies have revealed the mechanism of MET in the prevention and treatment of a variety of cancers, including breast, prostate, pancreatic, and lung cancers (Morales and Morris, 2015; Podhorecka et al., 2017; Saini and Yang, 2018). The most important mechanism is that it can reduce the expression of potential growth factors such as insulin and IGF-1, which can stimulate the survival and mitosis of tumor cells, and then inhibit the insulin-dependent mechanisms in the process of growth stimulation and metabolism. In addition, through the activation of the AMPK signaling pathway or by influencing chronic inflammation, cancer proliferation and progression of insulin-independent mechanisms are restricted (Morales and Morris, 2015). In a variety of cancer cell lines, MET can also increase the sensitivity of chemotherapy and reduce the side effects of chemotherapy, which can be used as adjuvant therapy combined with other drugs (such as adriamycin and PTX, etc.) (Morales and Morris, 2015). In addition, a large number of studies and clinical trials have proved that MET has good anticancer abilities alone and in combination. In recent years, the drug delivery system of MET has been designed with higher efficiency. Studies have shown that MET has a high affinity to serum albumin (Rahnama et al., 2015; Leboffe et al., 2020). Thus, MET-BSA NPs have been prepared with the weak binding of MET to BSA governed by hydrogen bonds and van der Waals forces using the anti-solvent precipitation method, which is a more effective therapeutic agent for liver tumor with insulin resistance than free MET (Lu et al., 2020). NPs have also been used to verify the strong association between type II diabetes and (MiaPaCa-2) pancreatic carcinoma cell lines (Jose et al., 2015). Here, the MET carrier has been widely studied as one of the most promising drug against pancreatic cancer, such as O-CMC-MET NPs based on MET-loaded O-CMC NPs. O-CMC-MET NPs can achieve a sustained release effect through the pH-dependent drug release, prolong the drug retention time in blood/circulation, and have blood compatibility; although it is nonspecific, due to the inhibition of mTOR activity in MiaPaCa-2 cells mediated by MET, the NPs showed a dose-dependent preferential toxicity to pancreatic cancer cells compared with normal cells (Snima et al., 2012). O-CMC-MET NPs induce apoptosis of MiaPaCa-2 cells mainly through the endogenous apoptotic pathway and affect cell cycle progression by downregulating the mRNA expression of p21, Vanin 1, and MMP9 (Snima et al., 2014). The optimized antitumor system of O-CMC-MET NPs is further embodied in breast cancer, which has better efficacy of the NPs compared with free MET for MCF-7 cells in a localized manner (De et al., 2019). MET-encapsulated positively charged liposomes (lipo-MET) could improve dose and activity of the drug, which significantly affected IC50 values, cell migration activity, colony formation, and apoptosis in MDA-MB-231 and MCF-7 cells (Shukla et al., 2019). Based on surface modification of the liposomes, herceptin-conjugated lipo-MET (Her-lipo-MET), as targeted liposomes with enhanced specificity, displayed better therapeutic efficacy than free MET or lipo-MET to the point of anti-proliferation and anti-migration in *in vitro* BCSC cells and increased the circulation of MET in the blood *in vivo*, resulting in enhancement of the anticancer effect. In particular, Her-lipo-MET further combining with DOX made these therapeutic efficacies more obvious (Lee et al., 2019). Lipo-MET also encapsulates VEGF-specific siRNAs to construct a potential dual-functional drug delivery system for combinational anti-oncogenic downregulation of the expression of VEGF and enhancing antitumor effect (Shi et al., 2017). A novel promising highly effective tumor therapy strategy has been fulfilled by loading novel photosensitizers (IR780 or IR775) into lipo-MET via photodynamic therapy (PDT) producing ROS to elicit more chemical damage of tumor cells (Yang et al., 2020; Xiong et al., 2021).

### 5-FU

5-FU is a fluoropyrimidine analog whose anticancer effects are mainly caused by the inhibition of thymidylate synthase (TS) and DNA synthesis and repair by directly incorporating its metabolites into the DNA and RNA of cancer cells. Owing to its low price and effective anticancer activity, 5-FU has been widely used alone or in combination with other anticancer drugs in the treatment of colorectal, breast, liver, pancreatic, esophageal, and gastric cancers, etc. (Wei et al., 2018). In general, 5-FU is administered intravenously, but its clinical application is limited by its unwanted side effects (such as hand and foot syndrome, mucositis/stomatitis, neutropenia, anemia, nausea/vomiting, and cardiotoxicity) and the low therapeutic response rate of cancer tissues. The poor accumulation of 5-FU in tumor tissues worsens this problem, and a high dosage has to be administered for expected efficacy. Thus, reducing the side effects by potentiating the anticancer activity of 5-FU or improving the accumulation of 5-FU in targeted regions seem applicable because a lower 5-FU dosage could be employed in clinical practice and the side effects could be accordingly alleviated. Various nanocarriers have been developed for encapsulating 5-FU with high loading and minimal side effects (Pan et al., 2017; Liu et al., 2018). 5-FU–loaded N, O-CMC NPs shows an initial burst release followed by a sustained release of 5-FU for a period of 48 h *in vitro* (Anitha et al., 2012a). The combinatorial nanomedicine of 5-FU and CUR could reduce the dose of 5-FU. The plasma half-life of 5-FU was improved in *in vivo* conditions up to 72 h (Anitha et al., 2014). FA-modified CMC-5-FU NPs microwave-induced through the skin and into melanoma cells were evaluated with respect to *in vitro* drug release, retention, and permeation, as well as *in vivo* pharmacokinetics profiles (Nawaz and Wong, 2018). FA-modified BSA NPs by carboxymethyl-β-cyclodextrin (CM-β-CD) had a high encapsulation efficiency for 5-FU. 5-Fu–loaded FA-CM-β-CD-BSA NPs were successfully constructed as good monodispersity, negative charge, and homogenous particle size enhancing FA receptor–mediated intracellular uptake of the drug and downregulating ATP levels and the expression of caspase-3 (Su C. et al., 2014). Also, 5-FU was loaded in FA-PEG-PAMAM (polyamidoamine) dendrimers educed hemolytic toxicity, which led to a sustained drug release pattern as well as highest accumulation in the tumor area in tumor-bearing mice (Singh et al., 2008). Chinnaiyan et al. (2019) prepared 5-FU–loaded Guar Gum–capped AuNPs (5FU-G-AuNPs) which exhibited potential cytotoxic and apoptotic effects on MiaPaCa-2 cell lines. Based on the combined and tumor-targeted PTT and chemotherapy, AuNPs coupled to 5-FU were photoactivated by near-infrared (NIR) to enable a spatial and temporal control of mild chemo-hyperthermia targeted to the tumor nodules within the peritoneal cavity which overcame the current off-target toxicity of hyperthermic intraperitoneal chemotherapy in clinical practice (Mulens-Arias et al., 2021). In order to enhance the efficacy and minimize the side effects of 5-FU, AgNPs were often conjugated with targeted agents such as FA or anti-EGFR antibodies, which exerted an effective targeted therapy for some cancers such as breast cancer, liver cancer, cholangiocarcinoma, and colorectal cancer, *in vivo* and *in vitro* (Ganeshkumar et al., 2014; Ngernyuang et al., 2016; Mani et al., 2018; Gajendiran et al., 2019; Liszbinski et al., 2020). Udofot et al. (2015) reported the cytotoxicity of 5-FU–loaded pH-sensitive liposomes in colorectal cancer cell lines. To enhance the antitumor efficacy of 5-FU against colorectal cancer, CS-coated flexible liposomes as a remarkable carrier were used to retard 5-FU release which was more effective in killing tumor cells in a sustained manner (Alomrani et al., 2019). Handali studied different targeted therapeutic strategies for colon cancer that liposomes encapsulating 5-FU mediated by different targeted ligands, such as FA and Tf could triggered the mitochondrial apoptotic pathway by decreasing the mitochondrial membrane potential, releasing of cytochrome c and promoting the substantial activity of caspase 3/7 (Handali et al., 2018; Moghimipour et al., 2018; Handali et al., 2019).

## Personalized Cancer Nanomedicine

**FIGURE 11:**
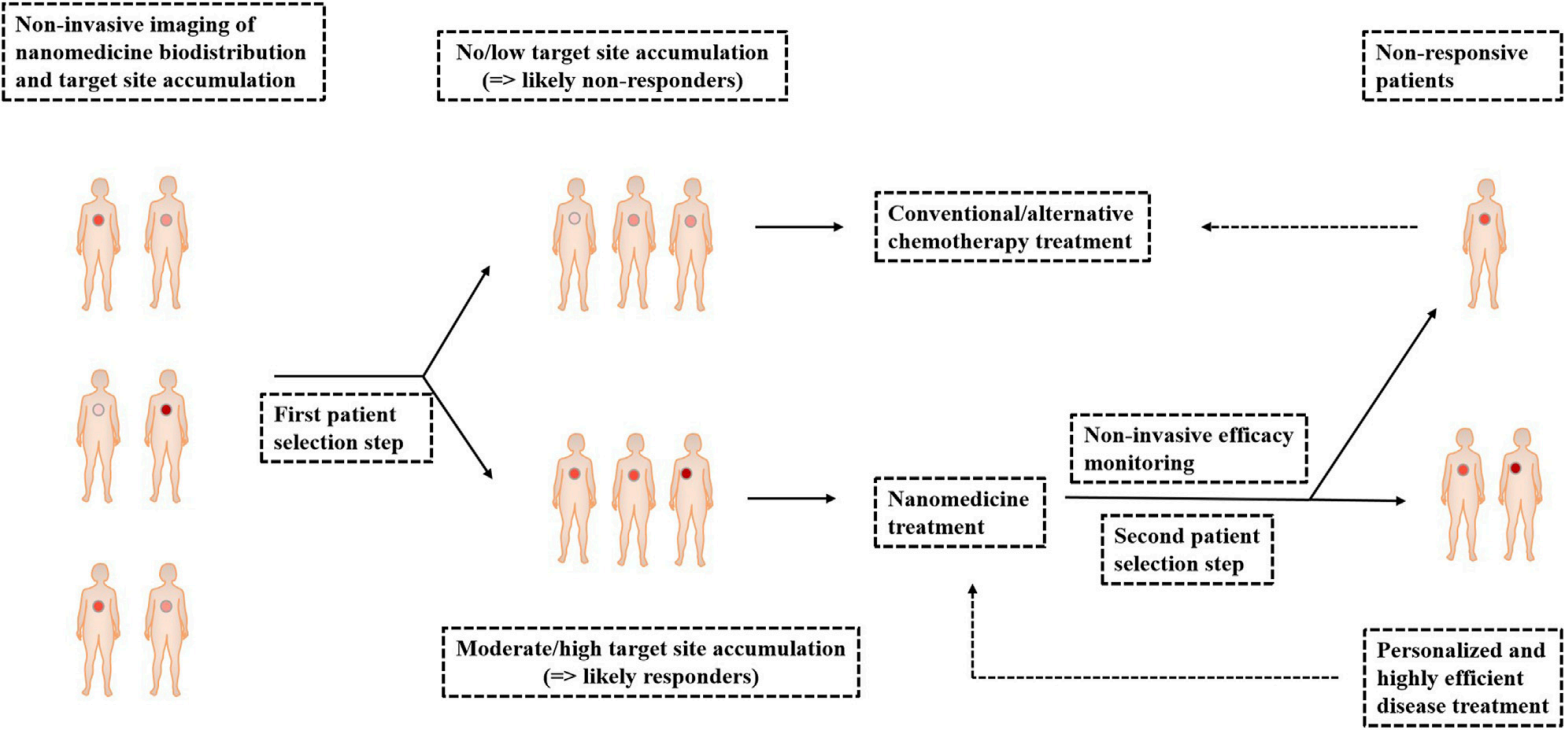
Personalized cancer nanomedicine. The nanomedicine biodistribution and target site accumulation of nanomedicine formulations labeled with contrast agents can preselect patients on the basis of noninvasive imaging (first patient selection step). And, then, the responders of moderate/high target site accumulation can either be assigned to nanomedicine treatment (second patient selection step). For the above two steps, the nonresponders of no/low target site accumulation and the nonresponsive patients of reasonable target site accumulation can be allocated to treatment with conventional/alternative chemotherapy to assure individualized and improved interventions: personalized and highly efficient disease treatment. Finally, nanomedicine treatment is repeatedly used for personalized and highly efficient disease treatment.

Personalized medicine is a strategy to achieve individualized and improved health care decisions via diagnosis, treatment, and monitoring of diseases and disease treatments which takes into account a single patient or single-disease unique profiles, including clinical, genomic, and environmental information, as well as the nature of diseases including their onset, course of progression, and response to treatment (Liu, 2012; Theek et al., 2014). In particular, personalized medicine is common in cancer therapy that is termed personalized oncology. The large intra- and intertumoral heterogeneity is the major hamper to accomplish effective cancer diagnosis and treatment. Versatile and adaptive materials and methods (like image-guided nanomedicine and targeted therapies) have been developed to evaluate these diverse individual tumors and cancer cells, as well as therapy characteristics in as much detail as possible (Liu, 2012; Theek et al., 2014). As shown in Figure 11, image-guided nanomedicine tracking drug delivery, drug release, and drug efficacy perform patient prescreening to identify amenable nanomedicine treatment for specific tumors. Nanomedicine biodistribution and target site accumulation seem to be highly useful to personalize tumor treatments (Lammers et al., 2012; Theek et al., 2014). Harrington et al. (2001) that indium-111–labeled PEGylated liposomes had significantly different degrees of EPR-mediated tumor accumulation between different types of tumors such as breast carcinoma, lung tumors, and neck carcinomas (Theek et al., 2014). Clinical case studies have shown that radiolabeled PEGylated liposomes accumulated highly efficiently in the primary tumor mass of a patient with Kaposi sarcoma, as well as in a number of secondary and/or metastatic lesions, and the patient had responded well to Doxil treatment with highly leaky, enhanced permeability and retention (Lammers et al., 2012). In other clinical trials, personalized nanomedicine based on nab-PTX has also been successfully applied to improve the response rates in breast cancer patients. And, in combination with gemcitabine, nab-PTX increased survival in patients with pancreatic cancer (Wicki et al., 2015). The development of personalized cancer therapeutics based on NPs revolutionizes the field of cancer therapy by significantly improving both the quality and duration of a patient's life (Sun et al., 2014).

## Conclusion and Expectation

Drug delivery systems are considered to be one of the most promising tools in cancer treatment, providing opportunities for complex multifunctional and targeted strategies. The most widely studied drug delivery systems include CMC NPs, BSA NPs, HFt NPs, liposomes, exosomes, dendrimers, PSiNPs, GO, AgNPs, and AuNPs. The development and application of these systems can increase the solubility and permeability of antitumor drugs, enhance the retention effect, prolong the circulation half-life, improve the biological distribution, and reduce the toxicity. Therefore, they have great research value in improving treatment strategies for cancer patients through personalized cancer nanomedicine. However, it is worth considering that most of these studies are in the early stage, and their clinical effects need to be further verified. Here, we analyze the drawbacks of MET, PTX, DTX, DOX, CUR, 5-FU, and siRNA alone and discuss the effective delivery and antitumor mechanism of drug delivery systems designed by these drugs, which may realize translating them from a preclinical level to the clinical setting.
